# Estimating the Cost-Effectiveness of Pre-Exposure Prophylaxis to Reduce HIV-1 and HSV-2 Incidence in HIV-Serodiscordant Couples in South Africa

**DOI:** 10.1371/journal.pone.0115511

**Published:** 2015-01-23

**Authors:** Britta L. Jewell, Ide Cremin, Michael Pickles, Connie Celum, Jared M. Baeten, Sinead Delany-Moretlwe, Timothy B. Hallett

**Affiliations:** 1 Department of Infectious Disease Epidemiology, Imperial College London, London, United Kingdom; 2 Departments of Global Health, Medicine and Epidemiology, University of Washington, Seattle, Washington, United States of America; 3 Wits Reproductive Health and HIV Institute, University of the Witwatersrand, Johannesburg, South Africa

## Abstract

**Objective:**

To estimate the cost-effectiveness of daily oral tenofovir-based PrEP, with a protective effect against HSV-2 as well as HIV-1, among HIV-1 serodiscordant couples in South Africa.

**Methods:**

We incorporated HSV-2 acquisition, transmission, and interaction with HIV-1 into a microsimulation model of heterosexual HIV-1 serodiscordant couples in South Africa, with use of PrEP for the HIV-1 uninfected partner prior to ART initiation for the HIV-1 1infected partner, and for one year thereafter.

**Results:**

We estimate the cost per disability-adjusted life-year (DALY) averted for two scenarios, one in which PrEP has no effect on reducing HSV-2 acquisition, and one in which there is a 33% reduction. After a twenty-year intervention, the cost per DALY averted is estimated to be $10,383 and $9,757, respectively – a 6% reduction, given the additional benefit of reduced HSV-2 acquisition. If all couples are discordant for both HIV-1 and HSV-2, the cost per DALY averted falls to $1,445, which shows that the impact is limited by HSV-2 concordance in couples.

**Conclusion:**

After a 20-year PrEP intervention, the cost per DALY averted with a reduction in HSV-2 is estimated to be modestly lower than without any effect, providing an increase of health benefits in addition to HIV-1 prevention at no extra cost. The small degree of the effect is in part due to a high prevalence of HSV-2 infection in HIV-1 serodiscordant couples in South Africa.

## Introduction

Pre-exposure prophylaxis (PrEP) has been demonstrated to be 44–75% effective at reducing acquisition of HIV-1 among uninfected individuals [1–5]. Two recent PrEP trials found that topical tenofovir gel and oral co-formulated tenofovir/emtricitabine (TDF/FTC) also decreased the acquisition of herpes simplex virus-2 (HSV-2) [1,4]. The CAPRISA 004 trial, which tested coitally-dependent use of a 1% tenofovir vaginal gel for HIV-1 prevention, found that the gel decreased HIV-1 incidence by 39% (95% confidence interval [CI] 6–60%), and HSV-2 incidence by 51% (95% CI 22–70%) [1]. Subsequently, the Partners PrEP Study found that daily oral TDF/FTC decreased acquisition of HIV-1 by 75% (95% CI 55–87%) and HSV-2 by 33% (95% CI 2–54%) in heterosexual HIV-serodiscordant couples [4,6,7]. HIV-1 serodiscordant couples have emerged as a potential key population for implementation of a PrEP intervention, given their sustained exposure to risk, need for additional prevention strategies in addition to condoms, and high adherence to PrEP [8].

In sub-Saharan Africa, high prevalence of both HIV-1 and HSV-2 infections has long been considered a factor in facilitating the transmission of the HIV epidemic [9–11]. HIV-1 and HSV-2 interactions are synergistic; the presence of one facilitates both the acquisition and onward transmission of the other [12,13]. Persons infected with HSV-2 are two to three times more susceptible to acquiring HIV-1, and HSV-2/HIV-1 co-infection is associated with higher HIV-1 infectiousness and faster HIV-1 disease progression [11,14]. Aside from the use of condoms, there are few effective primary prevention interventions to lower the risk of HSV-2 acquisition. Suppressive therapy for HSV-2 reduces the recurrence of genital ulcers and decreases transmission of HSV-2 in HIV-1 uninfected, HSV-2 serodiscordant couples by 48% [15], but the same effect was not observed in HIV-1 serodiscordant couples [16]. No HSV-2 vaccine has demonstrated efficacy in reducing HSV-2 acquisition [17]. Contracting HSV-2 can also have serious consequences for pregnant women; if acquired during the last trimester, the infection can be transmitted to the neonate during birth, and subsequently result in high rates of disability or the death of the infant [18]. This is particularly a concern in populations with high fertility rates in young women who are also susceptible to HSV-2 infection. The additional findings of the Partners PrEP Study on the HSV-2 effect therefore generated further enthusiasm that a PrEP intervention could increase health benefits for the same cost and potentially provide an unanticipated dual benefit by protecting against both HIV-1 and HSV-2.

We had previously modeled the impact and cost-effectiveness of oral PrEP for HIV-1 in South Africa, with no assumed effect on HSV-2 transmission [19]. This study found that a PrEP intervention among HIV-1 serodiscordant couples could be a cost-effective HIV-1 prevention strategy in South Africa. Given the observed efficacy of daily oral TDF/FTC PrEP against HSV-2, it is important to reassess the cost-effectiveness of PrEP in terms of disability-adjusted life-years (DALYs) based on both HIV-1 and HSV-2 efficacy. We provide revised estimates of the cost-effectiveness of PrEP for HIV-1 serodiscordant couples by incorporating the additional benefit of the protective effect of PrEP against HSV-2 acquisition.

## Methods

An existing microsimulation model [19] was revised to incorporate HSV-2 transmission, neonatal HSV-2 infection, and the interactions between HIV-1 and HSV-2 (Figure S1 in S1 Text), and was parameterized for South Africa [20]. Briefly, the model follows a set of heterosexual HIV-1 serodiscordant couples, and tracks progression of HIV-1, ART initiation, transmission of HIV-1 and HSV-2 within the couple–including to and from any external partners–and HSV-2 transmission from a woman to her infant at birth [18]. HSV-2 infection is assumed to have a constant low-level infectiousness that is not attenuated by ART [21]. The model is parameterized using data from the Partners in Prevention HIV/HSV Transmission trial, which took place among HIV-1 serodiscordant couples in 7 countries, including 3 sites in South Africa, from 2004–2008 [20,22]. In the trial, only couples in which the HIV-1 infected partner was also infected with HSV-2 were enrolled, and in 69% of trial couples, both partners were already infected with HSV-2. The model simulates only the trial couples, potentially neglecting a small sub-section of South African couples in which the HIV-1 infected partner is HSV-2 uninfected, in order to fit to the sex-specific HIV-1 and HSV-2 prevalence and incidence observed in the trial.

An intervention was simulated. From the time that the HIV-1 serodiscordant couple is “identified,” the HIV-1 infected partner in each couple initiates ART when their CD4 cell count falls below 350 cells/μl and the HIV-1-uninfected partner takes daily oral PrEP until their partner initiates ART and is assumed to achieve HIV-1 viral suppression (i.e. for one year following ART initiation), an approach which is consistent with current ART guidelines in South Africa. Intervention scenarios were compared to a baseline scenario of ART initiation at CD4 counts below 350 cells/μl, with no PrEP. DALYs were used to summarize health loss and gain, and can accrue through different stages of HIV-1 infection, HSV-2 infection, and the disability or death of infants as a result of neonatal HSV-2 [23,24]. A summary of key assumptions are available in Table 1, with further information about the model structure and parameters, including DALY weights, available in the online technical appendix (Tables S2-S3 in S1 Text). If an individual was infected with both HIV-1 and HSV-2, DALY weights for the respective stage of each disease were summed. The HIV-1 uninfected partner in the couple is assumed to be 90% adherent to PrEP, and TDF/FTC PrEP is assumed to be 90% efficacious against HIV-1 and 33% efficacious against HSV-2, giving an overall protective effect similar to that observed in the trial (Figure S2 in S1 Text). We assumed that efficacy of PrEP against HIV-1 was very high, given that PrEP efficacy with consistent adherence has been estimated at close to 100% in the iPrEx and Partners PrEP trials [3,25–27]. Serodiscordant couples observed in an adherence sub-study of the Partners PrEP trial also demonstrated very high adherence overall, and thus the functional effectiveness of PrEP in the model reflects observations from the trial [26].

**Table 1:** Key assumptions and parameters used in the model.

Parameter	Values	Source
Infectiousness of untreated individuals (relative to those with CD4 count ≥ 500 cells/μl	CD4 350–500: 1.00	Cohort of stable serodiscordant couples [34]
	CD4 200–350: 1.59	
	CD4 0–200: 4.99	
Mean time spent in CD4 cell count category (y)^a^	Infection to CD4 of 500: 2.4	Pooled analysis of African observational cohort studies [38]
	CD4 350–500: 2.4	
	CD4 200–350: 4.6	
	CD4 0–200: 2.6	
Relative infectiousness of those on ART (relative to those untreated with CD4 cell count <350 cells/μl)	0.08	Cohorts of stable serodiscordant couples [34,35]
Mortality rates on ART (per year)		Multiple observational cohort studies [39–41]
First year:		
ART initiation at CD4 500+	1.3%	
ART initiation at CD4 350–500	2.5%	
ART initiation at CD4 200–350	5%	
ART initiation at CD4 0–200	10%	
Subsequent years:		
ART initiation at CD4 500+	1.3%	
ART initiation at CD4 350–500	1.3%	
ART initiation at CD4 200–350	2.5%	
ART initiation at CD4 0–200	5%	
Drop-out from ART (per year)	First year: 10%; subsequent years: 5%	Observational data from programs in Zambia [42]
PrEP efficacy against HIV-1	90%	Consistent with the range of efficacy reported in PrEP trials after taking adherence into account [3,4,26]
PrEP efficacy against HSV-2	33%	Partners PrEP trial [7]
PrEP adherence	90%	Consistent with overall adherence reported in a sub-study of adherence in the Partners PrEP trial [26]
Multiplicative factor for increased susceptibility to HIV-1 if HSV-2 infection >1 year (prevalent HSV-2 infection)	3.0	Systematic review and meta-analysis of longitudinal studies [11]
Multiplicative factor for increased susceptibility to HIV-1 if HSV-2 infection <1 year (incident HSV-2 infection)	6.0	Assumed increase in susceptibility due to frequency of ulcers during primary HSV-2 infection [43–46]
Multiplicative factor for increased susceptibility to HSV-2 among those with HIV-1 infection	3.7	Cohort of adults in Uganda [13]
Multiplicative factor for increased transmission of HIV-1 among those with HIV-1/HSV-2 co-infection	3.0	Systematic review and meta-analysis of longitudinal studies [11]
Multiplicative factor for increased transmission of HSV-2 among those with HIV-1/HSV-2 co-infection	4.0	Cross-sectional study of HIV-1/HSV-2 co-infected women [47]
Relative reduction of acquisition of HIV-1 due to condoms per sex act, with respect to baseline transmission probability^b^	100%	Assumed
Relative reduction of acquisition of HSV-2 due to condoms per sex act, with respect to baseline transmission probability^b^	75%	[48]
Relative reduction of acquisition of HIV-1 due to circumcision per sex act, with respect to baseline transmission probability^b^	65%	[49]
Relative reduction of acquisition of HSV-2 due to circumcision per sex act, with respect to baseline transmission probability^b^	28%	Cohort of HIV-1 and HSV-2 uninfected men [50]
Probability of acquisition of neonatal HSV-2 if mother acquires HSV-2 in last trimester	33%	Cohort of pregnant women with HSV-2 infection [18]
Probability of acquisition of neonatal HSV-2 if mother’s HSV-2 is a reactivation	3%	Cohort of pregnant women with HSV-2 infection [18]
Probability of child death with neonatal HSV-2	65%	[51]
Given child survival, probability of child disability with neonatal HSV-2	80%	[51]
Full cost per year of ART	US $515	[52,53]
Full cost per year of PrEP	US $250	[19,54]

^a^Mean time elapsed between entering category (CD4 cell count reaching value of upper bound) and exiting category (CD4 cell count drops below value of lower bound).

^b^Baseline transmission probability is from an asymptomatic, non-pregnant woman to an uncircumcised man.

The calculation of the cost per DALY averted takes the perspective of the health care system (unless stated otherwise), in which gains from averted HIV-1 infections benefit the system by saving on later years of ART. Each result is the mean from a set of 100,000 simulated couples, and all costs and impacts are discounted at an annual rate of 3%.

## Results

**Figure 1:**
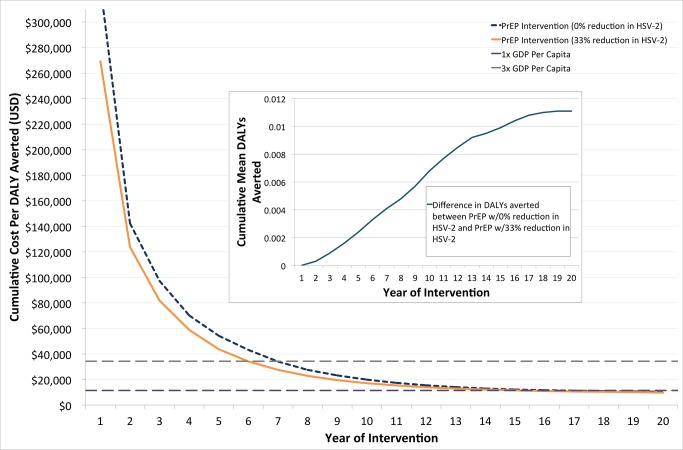
Difference in cost per DALY averted for two PrEP scenarios. The discounted cost per DALY averted for a 20-year PrEP intervention with no assumed protection against HSV-2 acquisition and with 33% protection (both relative to a baseline scenario of no PrEP and ART initiation at a CD4 count of 350 cells/μl). The inset is the difference between the two scenarios in the mean number of DALYs averted per couple over the intervention period. The horizontal lines represent WHO thresholds for cost-effectiveness at three times GDP ($34,320) and one times GDP ($11,440) for South Africa.

Fig. 1 shows the cost per DALY averted for the same PrEP intervention over a 20-year time horizon; the only difference is the effect of TDF/FTC PrEP on HSV-2 acquisition (0% or 33% efficacy). The cost per DALY averted after 20 years for a PrEP intervention with no assumed effect against HSV-2 is estimated at $10,383, and a 33% protection against HSV-2 yields an estimate of $9,757–a reduction of $626 (6%). Over the first seven years of the intervention, the cost per DALY averted drops dramatically for both scenarios, due to the accumulation of averted HIV-1 and HSV-2 infections. For PrEP with a 33% protective effect against HSV-2, the intervention is cost-effective according to the WHO’s cost-effectiveness threshold for three times GDP per capita after seven years, and for one times GDP per capita after 17 years [28]. DALYs related to neonatal HSV-2 make up less than 1% of total DALYs, and the benefit of reduced HSV-2 incidence averts 8% of DALYs related to neonatal HSV-2 compared to PrEP with no effect on HSV-2. The inset shows the difference in the number of averted DALYs between the two PrEP scenarios over time, with a greater differential towards the end of the hypothetical 20-year intervention period due to the accumulated benefit of averted HSV-2 infections and clinical consequences thereof. At the end of the 20-year intervention, however, the vast majority of the DALYs averted by the intervention originate from preventing new HIV-1 infections, and the overall added benefit of averting HSV-2 infections makes a minimal contribution.

**Figure 2:**
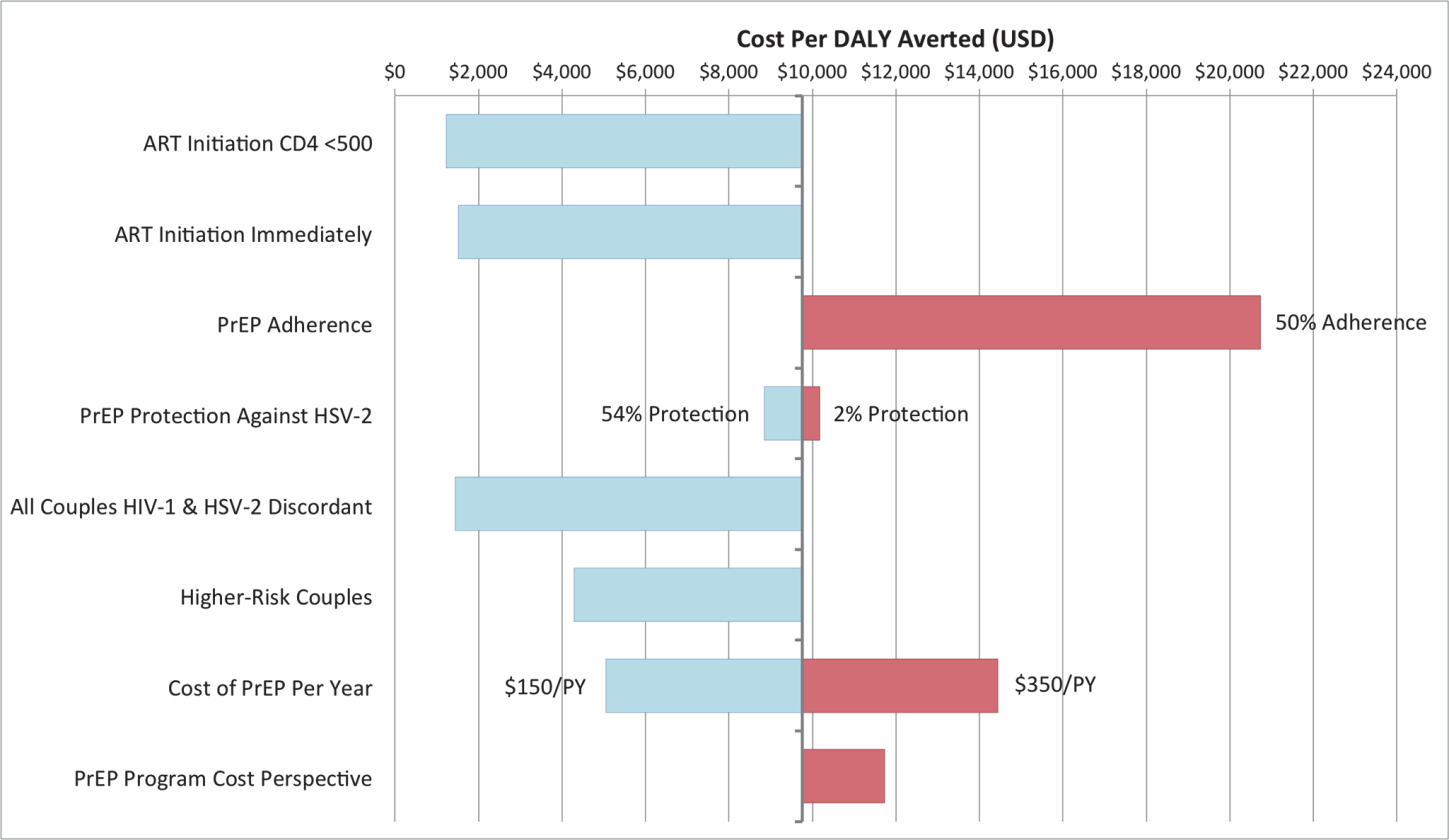
Sensitivity analysis for factors affecting the cost per DALY averted. Univariate sensitivity analysis for factors affecting the cost per DALY averted at the end of a 20-year PrEP intervention, with a baseline assumption of a 33% protection against acquisition of HSV-2 (the vertical line at $9,757). The bars titled *ART Initiation CD4 <500* and *ART Initiation Immediately* assume increased thresholds for ART initiation. The bar titled *PrEP Adherence* assumes HIV-uninfected individuals are 50% adherent to PrEP. The bar titled *PrEP Protection Against HSV-*2 explores the confidence intervals of the protective effect of HSV-2 from the Partners PrEP Study. The bar titled *All Couples HIV-1 & HSV-2 Discordant* simulates the same intervention among a set of couples in which one partner is dually infected with HIV-1 and HSV-2 and the other partner has neither infection. The bar titled *Higher-Risk Couples* assumes men are equally as likely to be the HIV-1 infected partner, condom use is reduced by 75%, 50% more couples have external partners, and the frequency of unprotected sex in external partners is doubled, in comparison to the demographic and behavioural characteristics of the South African HIV-1 serodiscordant couples who were enrolled in the Partners in Prevention HSV/HIV Transmission Study. The bar titled *Cost of PrEP Per Year* explores the cost per DALY averted if PrEP costs $150/PY or $350/PY, and the *PrEP Program Cost Perspective* bar assumes that the cost of the PrEP intervention is separate from funding for treatment, and does not include savings from reduced ART need due to averted HIV infections.

Fig. 2 shows univariate sensitivity analyses for several factors that contribute to uncertainty in the cost per DALY averted estimates, in which each factor is compared to a baseline cost of PrEP with an HSV-2 protective effect of 33%. The degree of the protective effect of PrEP against HSV-2, using the 95% confidence intervals from the Partners PrEP trial, has a minor additional impact on overall cost-effectiveness compared to other factors. Varying the protection against HSV-2 acquisition yields a range of $8,853–$10,355, for 54% and 2% efficacy, respectively. A lower cost per DALY averted are associated with increased thresholds for ART initiation, due to individuals spending less overall time on PrEP and greater ART savings from averted HIV-1 infections. PrEP can also be more cost-effective in scenarios in which all couples are dually discordant for HIV-1 and HSV-2–i.e. one partner has both infections and one has neither–or if the couples engage in higher-risk behaviors, e.g. reduced condom use and more unprotected sex. If PrEP is only taken during 50% of unprotected sex acts, the cost per DALY averted more than doubles, as the same number of person-years of PrEP are being used, but the impact is greatly reduced. Finally, if the intervention is funded separately to ART programs, the program is less cost-effective, and if PrEP costs are lower or higher than assumed, the cost per DALY averted changes linearly with respect to the price of PrEP. A multivariate sensitivity analysis was also carried out using Latin Hypercube Sampling, in which 400 parameter sets varying adherence to PrEP and the efficacy of PrEP on HSV-2 were evaluated in 24 different scenarios. This analysis yielded a spectrum of the cost per DALY averted ranging from a low of $486 per DALY averted to a high of $5.6 million per DALY averted (Table S4 in S1 Text). The highest costs per DALY averted resulted from extremely low adherence (2%), in which funds are being spent on PrEP with a negligible impact in terms of averted HIV-1 and HSV-2 infections. This wide interval suggests that the true cost per DALY averted for a hypothetical PrEP intervention in this population is uncertain.

## Discussion

Over a 20-year period, the efficacy of TDF/FTC PrEP to prevent new HIV-1 infections dominates the combined impact of PrEP on reducing HIV-1 and HSV-2 infections. The protective effect against HSV-2 has useful public health advantages, particularly given the lack of effective prevention strategies for HSV-2, but will not materially affect the cost-effectiveness of PrEP in HIV-1-serodiscordant couples. This is in part due to the relatively mild health consequences of HSV-2 in comparison to HIV-1; preventing HSV-2 incidence does not avert early death or years of severe morbidity in the same way that preventing acquisition of HIV-1 does. However, HSV-2 prevention is a potentially valuable supplementary benefit of PrEP, particularly in populations with lower HSV-2 prevalence than HIV-1 serodiscordant couples. Averting HSV-2 infections in women may also be especially valuable for the health system, given the risk of neonatal HSV-2 for pregnant women in their last trimester and the risk of serious morbidity and mortality for infants with primary HSV-2 infection. However, neonatal HSV-2 infection is a rare occurrence in itself, and the reduction in HSV-2 acquisition due to PrEP only protects a small fraction of women and their children from such an occurrence in a setting where HSV-2 prevalence is already high. Although HSV-2 treatment was not explicitly modeled in this analysis, the reduction in new HSV-2 infections may additionally benefit the health system by decreasing the need for HSV-2 treatment medications, such as acyclovir and valacyclovir.

In South Africa, as well as throughout sub-Saharan Africa, dual infection with HIV-1 and HSV-2 is high and acquisition of HSV-2 often occurs early in sexual activity, regardless of HIV-1 status [29,30]. HIV-1 serodiscordant couples are often identified after HSV-2 infection has already occurred (Table S1 in S1 Text) [22,31]; therefore, oral PrEP that provides partial efficacy against both HIV-1 and HSV-2 could demonstrate greater impact and improved cost-effectiveness in other populations. Primary prevention interventions like PrEP would have greater potential impact in reducing HSV-2 incidence in younger populations who have lower HSV-2 prevalence than serodiscordant couples. In the CAPRISA 004 trial among young women in South Africa, for example, incidence of HSV-2 was very high at 20.2 per 100 PY in the placebo arm [32], suggesting a greater opportunity for effective prevention of HSV-2 than in HIV-1 serodiscordant couples. A modelling study of the impact and cost-effectiveness of tenofovir gel among young women in Gauteng province in South Africa has also predicted that introducing coitally-dependent microbicide PrEP would be highly cost-effective, at less than $300 per DALY averted [33]. This intervention may be of more benefit in a population of young women simultaneously susceptible to HSV-2 and HIV-1 infection, and also at high risk of pregnancy. Although our analysis did not demonstrate a large effect in the cost-effectiveness of PrEP for HIV-1 serodiscordant couples, further modelling of oral PrEP is needed to investigate impact and cost-effectiveness in other populations, such as young women.

The cost per DALY averted is also dramatically reduced if the threshold for ART initiation is raised to CD4 counts <500 cells/μl or to immediate ART initiation upon a positive HIV-1 diagnosis. In these scenarios, HIV-1 uninfected partners in the couples spend less time on PrEP overall, and averted HIV-1 infections save a greater number of years of costly ART treatment. As early ART demonstrates greater cost-effectiveness than PrEP, earlier ART initiation may be preferable to a PrEP intervention in this population from a cost-effectiveness point of view. However, an important consideration for interventions in serodiscordant couples is the extent to which they can be considered “stable,” given that 25–30% of HIV transmission among couples has been shown to originate from an unlinked source [34,35]. If a substantial proportion of HIV-1 infections do indeed come from external partnerships, PrEP would be a preferable option to earlier ART. The preferences of couples themselves should also be taken into consideration, and some couples might choose to have the HIV-1 uninfected partner take PrEP, rather than earlier ART for the HIV-1 infected partner [36], especially if the couple is dually discordant for HIV-1 and HSV-2.

In South Africa, PrEP prioritized for serodiscordant couples could also make a useful contribution to HIV-1 prevention for less overall budgetary impact than early ART. In the South African sites in Partners in Prevention HIV/HSV Study, 27.4% of couples tested were HIV-1 serodiscordant [37], which may indicate hundreds of thousands of individuals for short-term PrEP use. Unlike early ART initiation, PrEP can be used as a prevention mechanism during “seasons of risk” only–e.g. during brief intervals of time during which the couple is trying to conceive and cannot use other prevention measures such as condoms–and does not necessarily require provision of costly medication for years. PrEP might be a useful addition to the combination prevention options currently available in South Africa, particularly in scenarios when earlier ART initiation means that fewer years of PrEP use are necessary. As with all cost-effectiveness analyses, our analysis does not consider affordability and it is not clear whether the WHO-recommended threshold represents the opportunity cost of displaced resources for health.

Ultimately, the additional benefit reaped by averting a small percentage of HSV-2 infections in HIV-1 serodiscordant couples leads to a modest decrease in the cost per DALY averted over a 20-year PrEP intervention. The magnitude of this benefit does not suggest a substantial departure from our previous understanding of the impact and cost-effectiveness of an oral PrEP intervention in this population, but may make such an intervention more appealing for HIV-1 serodiscordant couples–particularly those who are discordant for both HIV-1 and HSV-2–given this secondary beneficial effect.

## Supporting Information

S1 Text Model description, assumptions, and multivariate sensitivity analysis. (DOCX)
