# Supplementary material for: Estimating the Cost-Effectiveness of Pre-Exposure Prophylaxis to Reduce HIV-1 and HSV-2 Incidence in HIV-Serodiscordant Couples in South Africa
Source: PLoS One. 2015 Jan 23;10(1):e0115511. doi: 10.1371/journal.pone.0115511 (PMC4304839; doi:10.1371/journal.pone.0115511)
Supplement: S1 Text — (DOCX) [file pone.0115511.s001.docx]

**Technical Appendix**

**Model Structure**

The model is an individual-based simulation of HIV-1 and HSV-2 heterosexual transmission within South African HIV-1 serodiscordant couples, as well as to and from any external partnerships linked to the couple, in South Africa. A description of HIV-1 progression and calculation of transmission probability in the model has been provided previously [[1](#_ENREF_1)].

The model was updated to include acquisition and transmission of HSV-2 and the interaction between both infections, by including multiplicative factors for both transmissibility of and susceptibility to each infection. In addition, the previous model included HIV-1 transmission from external partnerships to the couple, but no transmission from the couple to external partners. The updated model tracks both HIV-1 and HSV-2 transmission to and from external partnerships, as well as transmission of neonatal HSV-2 to infants. As the model follows the number of DALYs over time, any disability that can cite the couple as its primary origin is included in the analysis.


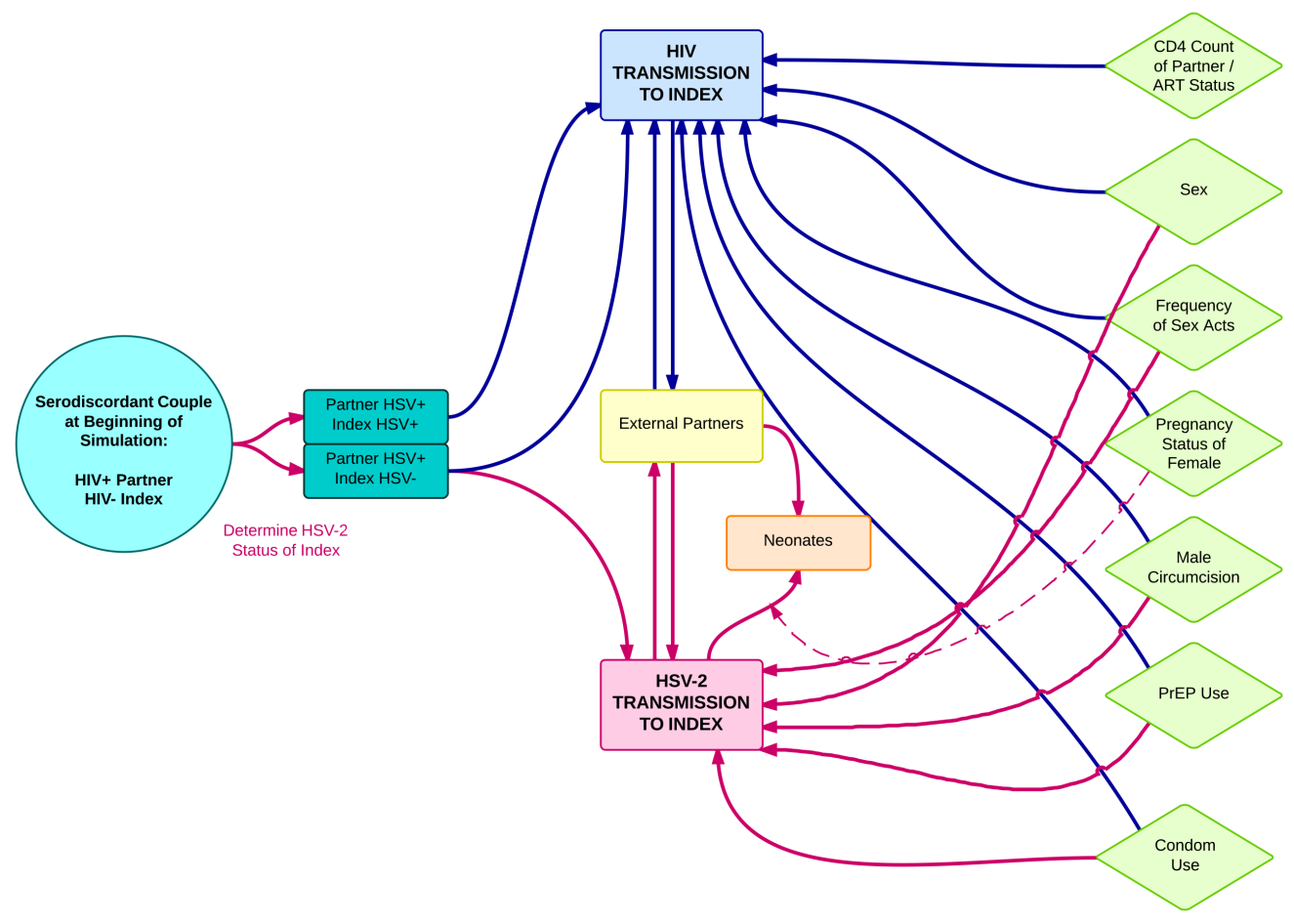


**Figure S1:** Diagram of HIV-1 and HSV-2 transmission within the model. Transmission for each infection is a function of factors listed in the green diamonds. The dotted pink line represents increased risk of HSV-2 transmission from a pregnant woman to the child if HSV-2 is contracted during her last trimester of pregnancy.

**Distribution of HIV-1/HSV-2 Co-infection**

|  | **Age-specific prevalence of HSV-2 (%)**  **Partners HSV/HIV Study, 2004-2008** | | | |
| --- | --- | --- | --- | --- |
|  | **18-24** | **25-34** | **35-44** | **44+** |
| **HIV-1 uninfected men in couple** | 28 | 53 | 65 | 73 |
| **HIV-1 uninfected women in couple** | 72 | 86 | 95 | 97 |

**Table S1:** Prevalence of HSV-2 with respect to age group, sex, and HIV-1 status from the Partners in Prevention HSV/HIV Study [[2](#_ENREF_2)]. All HIV+ partners were dually infected with HIV-1 and HSV-2.

**Model Assumptions**

Due to the frequency of asymptomatic shedding episodes of HSV-2, we assumed that HSV-2 can be represented as the transmission risk of an infection with constant low-level infectiousness. Implicitly, no change in sexual behaviour during symptomatic episodes is assumed for HSV-2 infected individuals. We do not explicitly model use of suppressive therapy (acyclovir or valacyclovir) for HSV-2 infections, given that use of acyclovir or valacyclovir for suppressive therapy for HSV-2 in South Africa is low [[3](#_ENREF_3)]. In addition, ART is assumed to have no effect on reducing incidence of HSV-2, as studies have not shown a decrease in asymptomatic shedding [[4](#_ENREF_4)].

PrEP is assumed to be 90% effective against HIV, 33% effective against HSV-2, and couples using PrEP are assumed to have 90% adherence to the drug. Efficacy of PrEP against HSV-2 and adherence are both varied in the univariate and multivariate sensitivity analyses. Couples are not assumed to increase risky sexual activity (risk compensation) as a consequence of PrEP use.

Both partners in the couple can acquire an external partner. A proportion of couples never have outside partnerships, while others begin and end partnerships to match a specific distribution at any point in time. The probability that an external partner is HIV-1 infected or HSV-2-infected is equivalent to the sex-specific prevalence of HIV-1 or HSV-2 in the general population, respectively, multiplied by a sex-specific parameter to adjust prevalence so as to generate incidence from outside partnerships that matches that seen in the trial. Either partner in the couple who has an external partner is assumed to have 6 unprotected sex acts with the external partner and 4 condom-protected sex acts. The number of protected acts is derived from the mean across the first month in stable partnerships, and the number of unprotected acts is assumed to be double that of stable partnerships in the first month. However, this choice of these values does not have a large influence over the results as the model is calibrated using separate risks of transmission for stable and external partners. If either the HIV-1 uninfected partner in the couple acquires HIV-1 as a consequence of the external partnership, or an HIV-1 uninfected external partner acquires HIV-1 from their partner in the couple, the course of the infection and resulting DALYs are tracked in the same way.

**Fitted Parameters**

The model was fit to two-year sex-specific HIV-1 and HSV-2 incidence rates from the Partners in Prevention HIV/HSV trial [[5](#_ENREF_5)]. Ten parameters (Table S2) were fit to the data. Fitting the model parameters was done simultaneously using a weighted least squares method, in which the goodness-of-fit statistic was weighted to person-years of observation. A best-fit parameter set was selected from 100,000 simulations.

| **Parameter** | **Fitted Value** | **Tolerated Bounds** |
| --- | --- | --- |
| Transmission probability of HIV-1 from an asymptomatic, non-pregnant woman to an uncircumcised man | **0.00014** | 0.0001-0.014 [[6](#_ENREF_6)] |
| Transmission probability of HSV-2 from an HIV-1 uninfected woman to an HIV-uninfected, uncircumcised man | **0.00025** | 0.0001-0.01 |
| Relative susceptibility of women (compared to men) at exposures to HIV-1 infection from stable partners (due to differences in condom use) | **0.25** | 0.25-2.0 [[7-9](#_ENREF_7)] |
| External partnership multiplier for women (due to underreporting) | **3.0** | 1.0-5.0 |
| External partnership multiplier for increased infectiousness of HIV-1 | **30.0** | 1.0-40.0 [[10](#_ENREF_10),[11](#_ENREF_11)] |
| Multiplier for increased likelihood of male external partners being infected with HIV-1 | **4.0** | 1.0-4.0 |
| Multiplier for increased likelihood of female external partners being infected with HIV-1 | **3.0** | 1.0-3.0 |
| Multiplier for increased likelihood of male external partners infected with HIV-1 also being infected with HSV-2 | **4.0** | 1.0-3.0 [[12](#_ENREF_12),[13](#_ENREF_13)] |
| Multiplier for increased likelihood of female external partners infected with HIV-1 also being infected with HSV-2 | **1.5** | 1.0-2.0 [[12](#_ENREF_12),[13](#_ENREF_13)] |
| Multiplier for male-to-female transmission of HSV-2 | **1.75** | 1.0-6.0 [[14](#_ENREF_14),[15](#_ENREF_15)] |

**Table S2:** Fitted parameters from the model, their fitted value, and the tolerated bounds for the fitting process.

Using the parameters in Table S2, Figure 2 shows the data and model comparison of HIV-1 and HSV-2 incidence.


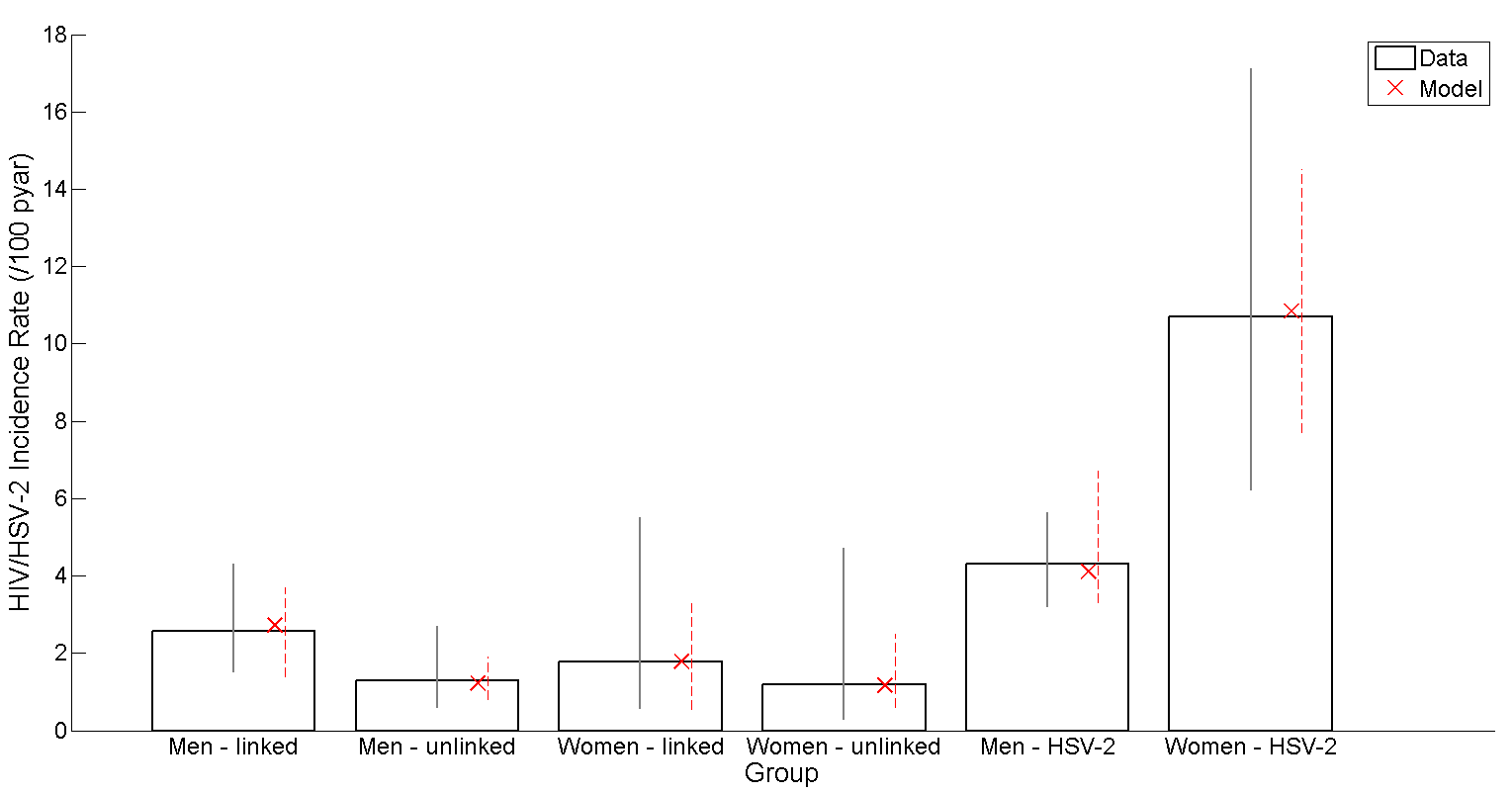


**Figure S2:** Comparison of Partners HIV/HSV trial data (bars) to model estimates (red crosses) of sex-specific HIV-1 and HSV-2 incidence within two years. Vertical grey lines represent confidence intervals from the data, and dashed red lines represent confidence intervals generated from the model. Transmission within the couple is represented as ‘linked’ and transmission from external partnerships is represented as ‘unlinked.’ For HSV-2, the source of transmission was undetermined in the trial, and model incidence is matched to overall HSV-2 incidence from both sources.

The basic transmission parameters for both HIV-1 and HSV-2 were fit while the multiplicative factors for HIV-1 and HSV-2 transmission remained static. We did not perform sensitivity analyses on these parameters, as the overall transmission parameters were adjusted for these parameters.

**Calculation of Cost-Effectiveness**

The cost-effectiveness of the PrEP intervention is calculated by comparing PrEP intervention scenarios to a baseline scenario in which the HIV-positive partner initiates ART once their CD4 count drops below 350 cells/µl. Each person-year of ART is assumed to cost $515 USD [[16](#_ENREF_16),[17](#_ENREF_17)], and each person-year of PrEP costs $250. ART cost was based on a weighted average of first and second line ARVs, service delivery and monitoring costs, and administrative and logistical costs. The cost of PrEP was based on a cost used in earlier studies [[1](#_ENREF_1),[18](#_ENREF_18)]. DALYs are accrued over time, as are person-years of PrEP and ART use, each with an annual discount rate of 3%.

**DALY Weights**

The DALY weights for HIV-1 are taken from the Global Burden of Disease Study 2010 [[19](#_ENREF_19)] and the value for HSV-2 from the Iran Burden of Disease and Injury Study 2003 [[20](#_ENREF_20)]. The Global Burden of Disease Study does not contain a DALY weight for HSV-2, but contains weights for sexually transmitted diseases that are not given specific weights. These values are equivalent to mild to moderate abdominopelvic conditions, with a weight in the range of 0.012-0.123; the estimate from the Iran Burden of Disease and Injury Study for HSV-2 falls in the middle of this range.

| **Health State** | **Disability Weight** | **Source** |
| --- | --- | --- |
| Herpes simplex virus-2 | 0.067 | Iran Burden of Disease and Injury Study, 2003 [[20](#_ENREF_20)] |
| HIV-1: CD4 count 500+, no ART | 0.051 | Global Burden of Disease Study 2010 [[19](#_ENREF_19)] |
| HIV-1: CD4 count 350-500, no ART | 0.051 | Global Burden of Disease Study 2010 [[19](#_ENREF_19)] |
| HIV-1: pre-AIDS, CD4 count 200-350, no ART | 0.221 | Global Burden of Disease Study 2010 [[19](#_ENREF_19)] |
| AIDS: CD4 count below 200, no ART | 0.547 | Global Burden of Disease Study 2010 [[19](#_ENREF_19)] |
| HIV-1/AIDS: receiving ART, first year | Assumed to be equivalent to disability weight at time of initiation | --- |
| HIV-1/AIDS: receiving ART, after first year | 0.053 | Global Burden of Disease Study 2010 [[19](#_ENREF_19)] |
| HIV-1: asymptomatic, CD4 count 500+ and HSV-2 | 0.118 | Addition of HIV-1 and HSV-2 weights |
| HIV-1: asymptomatic, CD4 count 350-500 and HSV-2 | 0.118 | Addition of HIV-1 and HSV-2 weights |
| HIV-1: pre-AIDS, symptomatic, CD4 count 200-350 and HSV-2 | 0.288 | Addition of HIV-1 and HSV-2 weights |
| AIDS: not receiving ART, CD4 count below 200 and HSV-2 | 0.610 | Addition of HIV-1 and HSV-2 weights |
| HIV-1/AIDS: receiving ART, first year and HSV-2 | Assumed to be equivalent to disability weight at time of initiation including HSV-2 weight | Addition of HIV-1 and HSV-2 weights |
| HIV-1/AIDS: receiving ART, after first year and HSV-2 | 0.120 | Addition of HIV-1 and HSV-2 weights |
| Neonatal death due to HSV-2 | 1, for a life expectancy of 60 years | Statistics South Africa [[21](#_ENREF_21)] |
| Child disability due to HSV-2 | 0.221 | Global Burden of Disease Study 2010 (motor plus cognitive impairments due to encephalitis) [[19](#_ENREF_19)] |

**Table S3:** List of DALY weights used in the model.

**Multivariate Sensitivity Analysis**

In order to fully explore the compounded effects of parameter choice on the cost-effectiveness of PrEP, multivariate sensitivity analyses were also conducted. Three parameters that were varied in the univariate sensitivity analyses in Figure 2 of the main text are continuous (adherence, efficacy of PrEP on HSV-2, and cost of PrEP), one is ternary (ART initiation threshold), and three are binary (discordancy status, risk behaviour, and the program cost perspective). In our analysis, we chose not to vary the cost of PrEP continuously, as it only changes the resulting cost per DALY averted linearly.

The analysis was carried out using Latin Hypercube Sampling (LHS), which allows the parameter space to be explored more efficiently than other more exhaustive methods of parameter variation. By forming a grid of *N* variables divided into *M* equally probably intervals, LHS samples each subsection of the grid only once. In this analysis, 24 separate combinations of the binary and ternary parameters (excluding the cost of PrEP) were possible. Using LHS, we selected twenty unique parameter values for both adherence to PrEP and the efficacy of PrEP on HSV-2, resulting in a total of 9,600 different parameter sets.

In the multivariate sensitivity analysis, the cost per DALY averted ranged from a low of $486 to a high of $5.6 million (Table S4). In scenarios with higher thresholds of ART initiation (<500 cells/µl and immediate ART), the difference between the lowest and highest cost per DALY averted is small, suggesting that adherence to PrEP and the efficacy of PrEP on HSV-2 are not driving the results to a great degree. In addition, if couples are dually discordant for HSV-2 and HIV-1 (i.e. one partner has both infections and the other partner has neither), less transmission of both HIV-1 and HSV-2 is occurring, and therefore the cost per DALY averted is not as strongly affected by low adherence or low efficacy of PrEP on HSV-2. If a PrEP program perspective is taken – i.e., costs for PrEP are separate to costs for ART – the cost per DALY averted is lower for earlier ART initiation scenarios, as there are many more years of costly ART being used for individuals who become infected. For scenarios in which ART initiation is <350 cells/µl, near-zero adherence plays the most significant role in determining the extremely high cost per DALY averted in four scenarios. These reflect a worst possible scenario and may be unrealistic. An important caveat for scenarios in which the highest cost per DALY averted is in millions of dollars is that extremely small numbers of DALYs are being averted, and thus the cost per DALY averted is sensitive to very minor changes in the number of DALYs averted. Lastly, if couples exhibit high-risk behaviours but have very low adherence, the ability of PrEP to avert more infections is outweighed by the lack of adherence.

| **ART Initiation Threshold (350 cells/µl, 500 cells/µl, Immediate)** | | | **Dually Discordant (Yes/No)** | **Higher-Risk Couples (Yes/No)** | **Program Cost Perspective (Yes/No)** | **Lowest Cost/DALY Averted** | **Highest Cost/DALY Averted** |
| --- | --- | --- | --- | --- | --- | --- | --- |
| 350 | 500 | Immediate |  |  |  | **$7,077** | **$1,794,936** |
| 350 | 500 | Immediate |  |  |  | **$1,343** | **$1,822** |
| 350 | 500 | Immediate |  |  |  | **$3,457** | **$5,613,708** |
| 350 | 500 | Immediate |  |  |  | **$8,673** | **$1,814,353** |
| 350 | 500 | Immediate |  |  |  | **$903** | **$1,560** |
| 350 | 500 | Immediate |  |  |  | **$1,783** | **$2,145** |
| 350 | 500 | Immediate |  |  |  | **$5,930** | **$5,685,551** |
| 350 | 500 | Immediate |  |  |  | **$1,632** | **$2,086** |
| 350 | 500 | Immediate |  |  |  | **$1,184** | **$1,447** |
| 350 | 500 | Immediate |  |  |  | **$752** | **$855** |
| 350 | 500 | Immediate |  |  |  | **$956** | **$1,288** |
| 350 | 500 | Immediate |  |  |  | **$871** | **$942** |
| 350 | 500 | Immediate |  |  |  | **$626** | **$782** |
| 350 | 500 | Immediate |  |  |  | **$614** | **$644** |
| 350 | 500 | Immediate |  |  |  | **$686** | **$769** |
| 350 | 500 | Immediate |  |  |  | **$539** | **$584** |
| 350 | 500 | Immediate |  |  |  | **$1,463** | **$1,768** |
| 350 | 500 | Immediate |  |  |  | **$924** | **$1,033** |
| 350 | 500 | Immediate |  |  |  | **$1,291** | **$1,672** |
| 350 | 500 | Immediate |  |  |  | **$800** | **$861** |
| 350 | 500 | Immediate |  |  |  | **$836** | **$1,005** |
| 350 | 500 | Immediate |  |  |  | **$557** | **$589** |
| 350 | 500 | Immediate |  |  |  | **$627** | **$699** |
| 350 | 500 | Immediate |  |  |  | **$486** | **$524** |

**Table S4:** Ranges of cost per DALY averted in each scenario evaluated in the multivariate sensitivity analysis. Green shading indicates which parameter values were being used in each scenario (“Yes”). Yellow shading indicates that those parameter values are not being used in the respective scenario (“No”). “Dually Discordant” indicates that all couples are composed of one HIV-1 and HSV-2 infected partner and one partner who is infected with neither HIV-1 nor HSV-2. “Higher-Risk Couples” indicates that couples have reduced condom use, more external partners, and increased frequency of unprotected sex with external partners. “Program Cost Perspective” indicates that PrEP program costs are separate to ART program costs, and does not include spending on ART.

**References for Technical Appendix**

1. Hallett TB, Baeten JM, Heffron R, Barnabas R, de Bruyn G, et al. (2011) Optimal uses of antiretrovirals for prevention in HIV-1 serodiscordant heterosexual couples in South Africa: a modelling study. PLoS Med 8: e1001123.

2. Lingappa JR, Kahle E, Mugo N, Mujugira A, Magaret A, et al. (2009) Characteristics of HIV-1 discordant couples enrolled in a trial of HSV-2 suppression to reduce HIV-1 transmission: the partners study. PLoS One 4: e5272.

3. Emge D, Delany-Moretlwe S, Mayaud P (2011) P5-S6.21 Acyclovir for managing genital ulcer disease in South Africa: experiences of guideline introduction, implementation and uptake. Sex Transm Infect 87: A334.

4. Tan DH, Kaul R, Raboud JM, Walmsley SL (2011) No impact of oral tenofovir disoproxil fumarate on herpes simplex virus shedding in HIV-infected adults. AIDS 25: 207-210.

5. Celum C, Wald A, Lingappa JR, Magaret AS, Wang RS, et al. (2010) Acyclovir and transmission of HIV-1 from persons infected with HIV-1 and HSV-2. N Engl J Med 362: 427-439.

6. Boily MC, Baggaley RF, Wang L, Masse B, White RG, et al. (2009) Heterosexual risk of HIV-1 infection per sexual act: systematic review and meta-analysis of observational studies. Lancet Infect Dis 9: 118-129.

7. Wawer MJ, Gray RH, Sewankambo NK, Serwadda D, Li X, et al. (2005) Rates of HIV-1 transmission per coital act, by stage of HIV-1 infection, in Rakai, Uganda. J Infect Dis 191: 1403-1409.

8. Padian NS, Shiboski SC, Jewell NP (1991) Female-to-male transmission of human immunodeficiency virus. JAMA 266: 1664-1667.

9. Nicolosi A, Correa Leite ML, Musicco M, Arici C, Gavazzeni G, et al. (1994) The efficiency of male-to-female and female-to-male sexual transmission of the human immunodeficiency virus: a study of 730 stable couples. Italian Study Group on HIV Heterosexual Transmission. Epidemiology 5: 570-575.

10. Rottingen JA, Cameron DW, Garnett GP (2001) A systematic review of the epidemiologic interactions between classic sexually transmitted diseases and HIV: how much really is known? Sex Transm Dis 28: 579-597.

11. Hollingsworth TD, Anderson RM, Fraser C (2008) HIV-1 transmission, by stage of infection. J Infect Dis 198: 687-693.

12. Auvert B, Ballard R, Campbell C, Carael M, Carton M, et al. (2001) HIV infection among youth in a South African mining town is associated with herpes simplex virus-2 seropositivity and sexual behaviour. AIDS 15: 885-898.

13. Weiss H (2004) Epidemiology of herpes simplex virus type 2 infection in the developing world. Herpes 11 Suppl 1: 24A-35A.

14. Wald A, Langenberg AG, Link K, Izu AE, Ashley R, et al. (2001) Effect of condoms on reducing the transmission of herpes simplex virus type 2 from men to women. JAMA 285: 3100-3106.

15. Langenberg AG, Corey L, Ashley RL, Leong WP, Straus SE (1999) A prospective study of new infections with herpes simplex virus type 1 and type 2. Chiron HSV Vaccine Study Group. N Engl J Med 341: 1432-1438.

16. World Health Organization (2011) Global HIV/AIDS response: epidemic update and health sector progress towards Universal Access. Geneva, Switzerland.

17. Menzies NA, Berruti AA, Blandford JM (2012) The determinants of HIV treatment costs in resource limited settings. PLoS One 7: e48726.

18. Gomez G, Borquez A, Case KK, Wheelock A, Vassall A, et al. (2013) The Cost and Impact of Scaling Up Pre-exposure Prophylaxis for HIV Prevention. PLoS Med 10: e1001401.

19. Murray CJ, Vos T, Lozano R, Naghavi M, Flaxman AD, et al. (2012) Disability-adjusted life years (DALYs) for 291 diseases and injuries in 21 regions, 1990-2010: a systematic analysis for the Global Burden of Disease Study 2010. Lancet 380: 2197-2223.

20. Naghavi M, Abolhassani F, Pourmalek F, Lakeh M, Jafari N, et al. (2009) The burden of disease and injury in Iran 2003. Popul Health Metr 7: 9.

21. Statistics South Africa (2013) Mid-year population estimates 2013.
